# Cavernomas: to treat or not to treat?

**DOI:** 10.1007/s00415-023-12115-0

**Published:** 2023-12-14

**Authors:** Sophie Voase, Neil P. Robertson

**Affiliations:** 1 Department of Neurology, University Hospital of Wales, Heath Park, Cardiff, CF14 4XN UK; 2 Institute of Psychological Medicine and Clinical Neuroscience, Cardiff University, University Hospital of Wales, Heath Park, Cardiff, CF14 4XN UK

## Introduction

Cerebral cavernous malformations (CCMs), also referred to as cavernomas or cavernous angiomas, can occur sporadically or as an inherited trait. CCMs can be asymptomatic and incidental findings on brain imaging or symptomatic because of haemorrhage, seizures, or headaches. Haemorrhage risk for CCMs vary according to whether they are incidentally detected and unruptured or have previously bled. The re-haemorrhage rate for CCMs is between 4.5 and 22.9% annually if there has been a single haemorrhage event, or up to 33% if there have been multiple previous events. Currently, there is no consensus on acute or longer term management of CCMs. In this month’s journal club, we explore three recent studies investigating interventions to reduce future haemorrhage risk and that propose future management strategies for symptomatic CCMs.

The first paper is a retrospective analysis of the efficacy of gamma knife stereotactic radiosurgery (GKSRS) for symptomatic CCMs from a single centre, and the second reports on the use and outcomes of stereotactic radiosurgery (SRS) in symptomatic CCMs from a series of international centres. The third paper reports on a clinical trial examining the use of propranolol in the treatment of familial symptomatic CCMs.

## Gamma Knife^®^ stereotactic radiosurgery for intracranial cavernous malformations

GKSRS is a technique in which SRS is delivered via a Gamma Knife model to CCMs. An Australian centre performed a retrospective review of 35 patients receiving GKSRS for symptomatic CCMs, with a minimum of 1-year follow-up. The authors collected data retrospectively, including location of CCM, number of lesions treated, pre-GKSRS haemorrhage, neurological deficits, seizures, and post-GKSRS events. They found a significant reduction in the matched annual haemorrhage rate (AHR) from pre-GKSRS (52.1%) compared to post-GKSRS (12.3%) (*p* < 0.001) [OR 0.07, 95% 0.008–0.283], but no statistically significant difference in seizure incidence pre-GKRS (30.7%) compared with post-GKSRS (17.9%) (*p* = 0.13) [OR 0.167, 95%CI 0.004–1.37]. One patient (3%) developed a long-term neurological deficit secondary to treatment-related oedema.

The same group also performed a systematic review and meta-analysis of current published evidence on SRS, totalling 1700 patients from 25 cohorts. This demonstrated a statistically significant reduction in relative risk of haemorrhage following SRS (random effects RR 0.12 (95% CI 0.074–0.198), *p* < 0.001). They also examined adverse events following SRS, finding a permanent adverse event rate of 4% (95% CI 2.8–5.8%) with 12.2% (95% CI 9–16.4%) experiencing any adverse effects.

### Comment

Re-haemorrhage in CCMs can leave lasting neurological deficits. This retrospective review suggests that GKSRS may be a well-tolerated and effective treatment in reducing re-haemorrhage risk in symptomatic CCMs. However, their findings suggest that GKRS may not influence the incidence of seizures. The findings are limited by the small cohort size and retrospective study design, which could introduce bias. The positive findings of this study suggest that a larger, randomised-controlled trial examining the use of SRS in the treatment of symptomatic CCMs is warranted.

*Shanker MD et al. J Clin Neurosci 2022; 106:96–102*. https://doi.org/10.1016/j.jocn.2022.10.015. *Epub 2022 Oct 20. PMID: 36,274,300.*

## Stereotactic radiosurgery for haemorrhagic cerebral cavernous malformation: a multi-institutional, retrospective study

SRS can be used for CCMs which are not amenable to surgical resection. This retrospective multi-centre study examined the effectiveness of SRS in symptomatic CCMs (defined as ≥ 1 haemorrhage event). There were 381 patients included in the study, with a pre-SRS AHR of 11.08 per 100 CCM-years, compared with a post-SRS AHR of 2.7 per 100 CCM-years. This was a statistically significant reduction in AHR post-SRS (− 8.33 per 100 CCM-years, 95% CI 6.67 to 10, *p* < 0.0001). Median follow-up of patients was greater than 3 years from SRS for imaging and clinical assessment. Adverse effects attributed to radiation occurred in 42 patients (11%), with greater volume of CCM > 0.7 cc (OR 5.19, 95% CI 2.41 to 12.5, *p* < 0.001) and margin dose > 13 Gy (OR 5.17, 95% CI 2.55 to 11.2, *p* < 0.001) found to confer an increased risk of adverse events. Almost a third of patients had improved neurological function at the last follow-up (119 out of 371).

### Comment

This retrospective multi-centre review gives further evidence that SRS may be beneficial in the management of CCMs which are not able to be resected surgically. This paper examined factors which increased the risk of adverse events, finding an association between higher incidence of adverse events and increased size of CCM and larger dose of radiation delivered. Future trials should factor these into the trial design to minimise the risk of adverse events. It remains unclear whether SRS is more effective than surgical excision in CCM management, so future trials should include two different treatment arms for resectable symptomatic CCMs.

*Dumot C et al. Stroke and vascular neurology 2023*. https://doi.org/10.1136/svn-2023-002380.

## Safety and efficacy of propranolol for treatment of familial cerebral cavernous malformations (Treat_CCM): a randomised, open-label, blinded-endpoint, phase 2 pilot trial

Observational studies and preclinical data suggested that beta-blockers may reduce the rate of intracerebral haemorrhage in symptomatic familial CCMs. This randomised, open-label, blinded-endpoint phase 2 pilot trial assigned patients to receive propranolol plus standard care (intervention group), or standard care alone (control group) for 24 months. The patients received 20–320 mg daily, according to how well they tolerated the doses and clinical assessments, and brain MRI were performed throughout the observation period. Patients were assessed for the primary outcome of new occurrence of symptomatic intracerebral haemorrhage or focal neurological deficit attributable to CCM over the period, as well as a secondary outcome of epileptic seizures.

The trial initially included 83 patients, of whom 57 were in the intervention group and 26 in the control group. In the intervention group, 3/57 discontinued (5%) propranolol due to side effects. After review, 12 patients were deemed ineligible due to the presence of sporadic CCM rather than familial CCM (9/12) or other MRI findings (3/12). Therefore, the total number of patients completing the trial was reduced to 67.

The incidence of new symptomatic haemorrhage or focal neurological deficit was 1·7 (95% CI 1·4–2·0) cases per 100 person-years (two [4%] of 57 participants) in the intervention group and 3·9 (3·1–4·7) per 100 person-years (two [8%] of 26) in the control group (univariable hazard ratio [HR] 0·43, 80% CI 0·18–0·98). As a result of inadequate power, the results were only able to demonstrate a signal towards efficacy for reduction in haemorrhage with propranolol treatment. The secondary outcome of seizures occurred in two (4%) participants in the intervention group (incidence 1·7 cases [95% CI 1·3–2·0] per 100 person-years) and one (4%) in the control group (1·9 [1·4–2·5] per 100 person-years; HR 0·92 [95% CI 0·08–10·12]). An MRI sub-study of 67 participants was conducted which found a median number of four d*e novo* CCMs in the intervention group versus five in the control group.

### Comment

This trial was not adequately powered to investigate the efficacy of propranolol compared with standard treatment or the effect of different doses on the incidence of clinical events. However, it offers some promise that propranolol could reduce the incidence of new symptomatic haemorrhage events in people with symptomatic familial CCMs but may not influence seizures associated with CCMs. The trial was also not adequately powered to assess the effect of medical management on formation of de novo CCMs. Further trials with a larger sample size and longer follow-up period are clearly required to demonstrate efficacy. Future trials should also examine any difference in the effect of treatments on familial and non-familial CCMs.

*Lanfranconi S et al. The Lancet. Neurology 2023; 22(1):35–44*. https://doi.org/10.1016/S1474-4422(22)00409-4.

## Conclusion

This month’s journal club explored three recent papers on the topic of symptomatic cerebral cavernous malformations, with two positive outcomes due to a reduction in re-haemorrhage rate with SRS and one which suggests a positive effect of medical management in symptomatic CCMs. As symptomatic CCMs are relatively rare, single-centre studies are unlikely to obtain large cohort sizes and, therefore, a multi-centre approach is required. Future trials should focus on a comparison of medical versus surgical management of symptomatic CCMs, as well as differences between surgical excision and SRS. Future trials should also examine differences in re-haemorrhage rates by location of CCM and the efficacy of treatment strategies by location.

